# From Black Box to Biological Insight: AttentioFuse Unlocks Multi-Omics Dynamics in Lung Cancer

**DOI:** 10.3390/cancers18050878

**Published:** 2026-03-09

**Authors:** Yuhang Huang, Yungang He, Liyan Zeng, Lei Liu, Fan Zhong

**Affiliations:** 1 Institutes of Biomedical Sciences, Fudan University, 131 Dongan Road, Shanghai 200032, China; 2 Intelligent Medicine Institute, Fudan University, Shanghai 200032, China; 3 Shanghai Institute of Infectious Disease and Biosecurity, Fudan University, Shanghai 200032, China; 4 Shanghai Institute of Stem Cell Research and Clinical Translation, Shanghai 200120, China

**Keywords:** interpretable deep learning, mid-fusion, multi-omics, hierarchical attention, NSCLC

## Abstract

Background: Lung adenocarcinoma (LUAD) and squamous cell carcinoma (LUSC), the major subtypes of non-small cell lung cancer (NSCLC), exhibit distinct molecular landscapes that demand precision in prognosis and therapy. While deep learning models can achieve high predictive accuracy, their black-box nature limits clinical translation. Methods: We introduce AttentioFuse, an interpretable deep learning framework employing a Reactome-guided mid-fusion strategy for multi-omics integration. AttentioFuse builds on three pillars: (i) dual-phase learning with omics-specific encoders to preserve modality-unique patterns, (ii) hierarchical attention mechanisms (cross-omics, feature-level, and fusion-layer) to quantify layer contributions dynamically, and (iii) integrated explainability combining DeepSHAP and global attention weights for gene-to-pathway interpretation. Two depth variants are instantiated under identical priors: a three-layer configuration (3F) for main discrimination and a five-layer configuration (AttentioFuse-5X) for deeper hierarchical interpretation; the 5X variant is trained end-to-end and yields comparable accuracy while enhancing pathway-level resolution. Results: Evaluated on The Cancer Genome Atlas (TCGA) LUAD/LUSC cohorts, AttentioFuse matches state-of-the-art performance in TNM staging while uncovering actionable biological insights, including pan-NSCLC AKT/mTOR metabolic control, histology-divergent Notch signaling roles, and additional pathways related to developmental reactivation, microbiota-associated metastasis, and extracellular matrix remodeling. Conclusions: By design, AttentioFuse-5X bridges predictive performance with hierarchical, pathway-resolved explanations, advancing oncology by transforming black-box predictions into biologically grounded decision support.

## Simple Summary

Lung cancer is often staged using imaging and pathology, but tumors within the same stage can behave very differently. Multi-omics profiles contain complementary information that may improve risk stratification, yet many deep learning models act as “black boxes” that are hard to audit clinically. We present AttentioFuse, an interpretable Reactome-guided mid-fusion framework that links predictive signals to a gene–pathway–modality evidence chain. In TCGA LUAD/LUSC cohorts, AttentioFuse achieves competitive performance for T- and N-stage prediction and provides pathway-resolved explanations that are coherent across folds. Importantly, these explanations are hypothesis-generating and do not imply causality without experimental validation.

## 1. Introduction

Non-small cell lung cancer (NSCLC) is dominated by LUAD and LUSC, two histologies that together account for over 80% of cases yet differ markedly in molecular circuitry and clinical behavior [1]. While tumor-node-metastasis (TNM) remains the clinical backbone for staging, labels are influenced by imaging/pathology workflows and can be noisy across centers, complicating the development—and interpretation—of purely molecular predictors.

Recent progress in artificial intelligence (AI), machine learning (ML), and deep learning (DL) has enabled predictive modeling from high-dimensional molecular measurements and clinical endpoints. In oncology, such models are increasingly explored for patient stratification, biomarker discovery, and treatment-related decision support, especially when informative signals are distributed across heterogeneous omics layers. However, clinical translation typically requires more than headline accuracy: models should be auditable, mechanism-linked, and robust to cohort and workflow heterogeneity [2,3,4].

Multi-omics integration (transcriptome, copy-number variation, single-nucleotide variants, etc.) is a natural route to expose mechanisms underpinning prognosis and treatment response. Deep models often achieve strong discrimination, but their black-box nature limits clinical translation where mechanism-aware evidence is required [5]. Integration strategies span early fusion (IntegratalNet; concatenate then encode), mid-fusion (independent encoders with intermediate interaction), and late/ensemble fusion (decision-level aggregation). Beyond generic fusion, biologically informed networks align hidden units to pathway hierarchies to yield pathway-level attributions (e.g., PASNet; P-NET as a mask-based variant) [6,7,8,9,10]. Parallel lines include factor/similarity approaches (MOFA, DIABLO, SNF) that learn shared/omics-specific structure [11,12,13], and recent attention-based multimodal fusion that provides modality-level weights and better tolerance to missing modalities [14,15].

Recent technical reviews provide systematic taxonomies of multi-omics integration methods, spanning classical correlation/matrix-factorization approaches to modern deep learning and deep generative architectures, and emphasize practical issues such as missing modalities, normalization, and cohort shift. These overviews motivate mid-fusion designs that preserve modality-specific encoders while explicitly modeling cross-omics interactions [16,17,18].

In parallel, there is growing emphasis on explainable AI (XAI) for omics modeling, where explanations should be reproducible across resampling or retraining and should connect feature-level signals to biologically meaningful units (e.g., pathways). Pathway-guided interpretable deep learning architectures have therefore become an active direction for reconciling predictive modeling with mechanism-oriented interpretation [19,20].

Under real-world constraints, heterogeneous cohorts pose practical issues we do not claim to eliminate: label-uncertainty in morphology-driven TNM; incomplete priors, as curation under-represents many regulatory programs; and cross-cohort heterogeneity in assay pipelines and omics availability (e.g., TCGA has SNV/CNV/transcriptome, whereas external resources may offer only a subset). Accordingly, auditability is emphasized alongside headline accuracy, and external cohorts are treated as feasibility checks under available modalities.

Although pathologic TNM is determined post-operatively, multi-omics profiles can be obtained from pre-treatment biopsies or diagnostic specimens. Our goal is therefore not to replace pathology, but to provide preoperative, mechanism-linked risk stratification that complements imaging-based staging when uncertainty exists. In the clinical timeline, the proposed prediction can be applied (i) after diagnostic sampling but before definitive therapy, to flag patients likely to harbor locally advanced disease (T3–4/N1–3) or distant spread (M1) and thus motivate intensified staging work-up and multidisciplinary planning; and (ii) perioperatively, to contextualize molecular aggressiveness and guide hypothesis-driven discussions of neoadjuvant/adjuvant strategies. Because TNM labels can be workflow-dependent and noisy across centers, we adopt clinically interpretable binarizations (Tis/T1–2 vs. T3–4; N0 vs. N1–3; M0 vs. M1) to reduce label granularity-driven variance while preserving decision-relevant thresholds.

**Figure 1:**
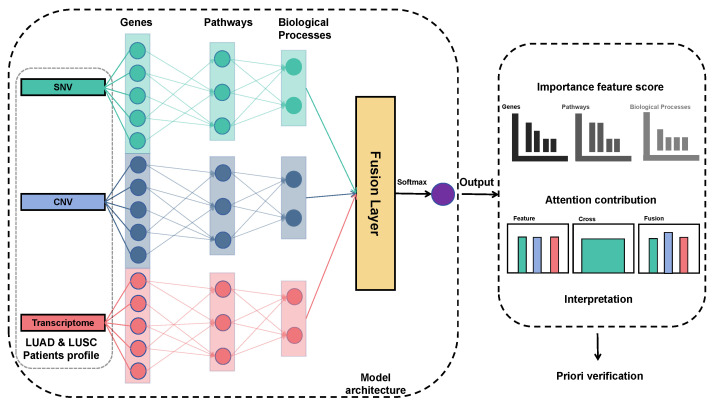
Architecture of the AttentioFuse framework. The framework processes multi-omics data (single-nucleotide variants (SNV), copy-number variation (CNV), and transcriptome) through individual sub-networks to preserve omics-specific patterns. These representations are fused at a mid-stage using a trainable attention layer, weighting each omics layer’s contribution. Interpretation combines DeepSHAP with global attention weights, providing feature-importance scores at gene/omics levels and attention contribution visualizations. A five-layer variant (AttentioFuse-5X) extends the masked hierarchy to deepen pathway granularity while retaining the same priors and fusion design.

AttentioFuse is an interpretation-oriented mid-fusion framework with (i) omics-specific encoders to preserve modality-unique signals, (ii) a hierarchical attention stack (feature-, cross-omics-, fusion-level) that yields sample-wise modality weights and pathway contributions, and (iii) integrated explainability that reconciles local DeepSHAP gene attributions with global attention summaries into a consistent gene-to-pathway-to-omics evidence chain [21]. AttentioFuse is presented in two depth variants (3F and 5X); the five-layer AttentioFuse-5X emphasizes hierarchical interpretability under end-to-end fine-tuning without materially altering model ranking. By design, the architecture remains modular when some modalities are absent (Figure 1).

Compared with prior multi-omics predictors, AttentioFuse is designed to address three gaps simultaneously: (i) preserving modality-specific structure via masked omics encoders while enabling explicit cross-omics interaction modeling at a mid-fusion stage; (ii) producing a coherent gene-to-pathway evidence chain by combining gene-level attributions with pathway-aware aggregation under Reactome priors; and (iii) offering a dual-depth instantiation under identical priors, where 3F serves as the main benchmarking backbone and 5X increases hierarchical resolution for interpretation without aiming for performance gains.

Preview on LUSC and LUAD. In LUSC-only previews, AttentioFuse-3F attains ≈0.92 accuracy (ACC)/≈0.91 F1 on T-stage and ≈0.86/≈0.85 on N-stage without class balancing, exceeding early-fusion counterparts; M-stage saturates across models at ≈0.98. In LUAD-only previews, AttentioFuse-3F reaches ≈0.91/≈0.90 on T-stage and ≈0.86–0.87 on N-stage, while M-stage is near-ceiling (≈0.93–0.97). These trends persist with or without SMOTE—balancing shifts absolute numbers slightly but does not change relative ordering [22].

Our goal is mechanism-aware decision support under realistic constraints: deliver competitive discrimination and surface pathway-resolved rationales, as well as make explicit where priors, labels, and modality availability cap performance. Alongside discrimination, we report calibration and decision-analytic summaries to contextualize utility, and we quantify explanation stability.

## 2. Materials and Methods

### 2.1. Data Acquisition and Processing

The primary cohort was TCGA LUAD/LUSC, accessed via TCGAbiolinks (downloaded September 2024) [23]. mRNA expression matrices were processed by removing mitochondrial genes (MT-*) and applying log1p; gene-level CNV followed GISTIC2, with high-confidence events retained [24]. SNVs were derived from MAF-like inputs by excluding non-functional classes (silent/UTR/intron/IGR), applying a t_alt_count > 5 filter when available (otherwise skipped with logging), deduplicating sample–gene pairs, and pivoting to a binary sample × gene mutation matrix (any qualifying variant to 1). All omics were harmonized to 12-character TCGA short IDs with duplicate aliquots averaged.

Pathologic TNM labels were binarized into clinically interpretable groups—T (Tis/T1–2 vs. T3–4), N (N0 vs. N1–3), and M (M0 vs. M1) [25]—to reduce label-granularity-driven variance and mitigate workflow-related label noise while preserving clinically actionable decision thresholds. Clinical rows were deduplicated by patient identifier; TX/NX/MX and not reported were excluded. For each analysis we aligned patients by short-ID intersection across the required modalities and re-indexed matrices accordingly. To avoid leakage, normalization and fold-wise feature-mean imputation were fit on training folds and applied to validation/test portions using training statistics only. Class imbalance was probed via an optional borderline-SMOTE (Borderline Synthetic Minority Over-sampling Technique, Borderline-SMOTE) (k = 5, capped at ≤15% synthetic samples) applied within training folds; we report results with and without this option.

External evaluation was conducted as a portability feasibility check using publicly available resources under a modality-available protocol [26,27,28]. Because external resources may differ in assay pipelines and often provide only a subset of modalities, external experiments were restricted to the modalities available in each dataset and are interpreted as generalizability signals rather than head-to-head biological comparisons. Harmonization followed consistent steps: identifier standardization and gene-name cleaning (including removal of version suffixes where applicable), feature-space alignment by intersection to a shared gene set, and within-cohort preprocessing (numeric coercion, imputation of missing values where needed, and scaling). When preprocessing parameters were transferred, transformations were fitted using training data only and applied to held-out splits without accessing external labels.

Overall, these external analyses suggest that AttentioFuse can qualitatively transfer under modality-available settings, while performance is primarily constrained by domain shift and missing modalities rather than the fusion design itself; full external portability results are provided in Supplementary Material File S1.

We assembled TCGA LUAD and LUSC multi-omics cohorts by harmonizing patient identifiers (12-character TCGA short IDs) and retaining only patients present in all required modalities. This yielded 505 LUAD and 482 LUSC patients with tri-omics profiles (mRNA, CNV, and SNV). For each staging task, we then excluded cases with missing or unknown pathologic labels (TX/NX/MX or not reported) and applied the predefined binarizations (Tis/T1–2 vs. T3–4; N0 vs. N1–3; M0 vs. M1). As a result, the final label-available sample sizes were: LUAD—T: 503 (438/65), N: 494 (326/168), M: 366 (341/25); and LUSC—T: 407 (333/74), N: 400 (263/137), M: 334 (329/5). Counts are reported as class 0/class 1.

### 2.2. Model Design

**Figure 2:**
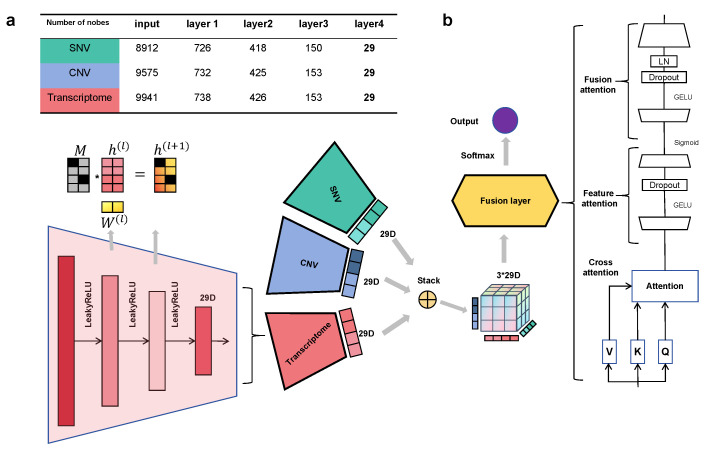
AttentioFuse model architecture and interpretation methodology. (**a**) Node counts per masked layer for each omics-specific sub-network in the AttentioFuse-5X variant; AttentioFuse-3F follows the same Reactome-based hierarchy at reduced depth. (**b**) Mid-fusion stage, showing stacked omics representations, the fusion layer with attention mechanisms, and the interpretation workflow.

AttentioFuse employs mask matrices derived from the Reactome database (v86) [29] to constrain layer connectivity. We instantiate two depth variants that share the same Reactome priors: a three-layer configuration (3F) used for main classification experiments, and a five-layer configuration (5X) used when deeper pathway hierarchy and interpretation are the focus. The 5X shares identical Reactome masks and yields comparable predictive accuracy but introduces extra depth and parameters primarily useful for hierarchical interpretation (Figure 2).

#### 2.2.1. Omics-Specific Encoders

Each molecular modality is processed through dedicated pathways using masked linear layers:

(1) $$h_{m}^{\left(\ell +1\right)}=\phi \;\left(\left(W_{m}^{\left(\ell \right)}⊙M^{\left(\ell \right)}\right)h_{m}^{\left(\ell \right)}+b_{m}^{\left(\ell \right)}\right),$$

where $M^{\left(\ell \right)}$ denotes Reactome-derived binary masks, ⊙ is the Hadamard product, and ϕ(·) is LeakyReLU with α=0.01:

(2) $$\mathrm{LeakyReLU}\left(x\right)=\left\{\begin{array}{cc} x, & x>0,\\ \alpha x, & x\leq 0. \end{array}\right.$$

Intuitively, each masked layer propagates signals only along Reactome-supported gene–pathway links, so the model learns pathway-structured representations rather than unconstrained dense mappings.

Gene–pathway relationships from Reactome were encoded as binary mask matrices. During training, these masks were applied to W via Hadamard product, suppressing weights of Reactome-unsupported connections and enforcing the network to learn biologically supported gene–pathway relationships.

#### 2.2.2. Cross-Omics Attention Fusion

The fusion module processes multi-omics embeddings (m modalities; e.g., m = 3 for TCGA: mRNA/CNV/SNV) through a hierarchical attention mechanism [30]. Each modality is encoded into a 29-dimensional pathway activation vector by the Reactome-guided encoders. The multimodal integration module employs attention to dynamically weigh cross-omics interactions:

(3) $$\mathrm{Attn}\left(Q,K,V\right)=\mathrm{softmax}\;\left(\frac{QK^{⊤}}{\sqrt{d}}\right)V,$$

where Q,K,V are linear projections of the tri-omics embeddings and *d* is the pathway dimension for scaling.

Here, the attention weights quantify how strongly one omics embedding attends to another, enabling sample-specific cross-omics interaction modeling.

A two-layer neural gate *g* dynamically weights combined features:

(4) $$g=\sigma \;\left(W_{2}\;\mathrm{GELU}\;(\mathrm{Gaussian}\;\mathrm{Error}\;\mathrm{Linear}\;\mathrm{Unit})\;\left(W_{1}z+b_{1}\right)+b_{2}\right),$$

where σ is the sigmoid function and z stacks modality-pathway features. This gate acts as a soft feature selector, amplifying informative fused components while down-weighting less relevant ones. The final fused embedding integrates gated features while preserving original modality characteristics through residual learning:

(5) $$h_{\mathrm{fuse}}=W_{f}\;\left(g⊙z\right)+\frac{1}{m}\sum_{i=1}^{m}h_{i}.$$

The residual term preserves the average modality signal, which stabilizes training and avoids over-reliance on a single omics layer.

#### 2.2.3. Interpretation Methods

For a sample *x* and modality *m*, DeepSHAP yields gene-level attributions $\phi_{m,g}\left(x\right)$ for each input gene *g* (signed contributions to the logit of the positive class). We define the global gene importance as

(6) $$I_{m,g}^{\mathrm{gene}}=\frac{1}{\left|D\right|}\sum_{x\in D}\left|\phi_{m,g}\left(x\right)\right|.$$

We use the mean absolute attribution so that both positive and negative contributions are treated as evidence strength.

Let $A_{g,p}\in \left\{0,1\right\}$ denote Reactome membership (gene *g* belongs to pathway node *p* under the masked hierarchy). We aggregate gene attributions to modality-specific pathway scores:

(7) $$S_{m,p}\left(x\right)=\sum_{g}A_{g,p}\;\phi_{m,g}\left(x\right),\;I_{m,p}^{\mathrm{path}}=\frac{1}{\left|D\right|}\sum_{x\in D}\left|S_{m,p}\left(x\right)\right|.$$

This step aggregates gene-level attributions to the pathway level according to Reactome membership, yielding pathway-resolved evidence within each modality.

From the fusion module we extract attention-derived modality weights $\alpha_{m}\left(x\right)$, normalized by $\sum_{m}\alpha_{m}\left(x\right)=1$. The integrated pathway importance is then

(8) $$I_{p}^{\mathrm{int}}=\frac{1}{\left|D\right|}\sum_{x\in D}\left|\sum_{m}\alpha_{m}\left(x\right)\;S_{m,p}\left(x\right)\right|.$$

This integrates pathway scores across modalities using attention-derived modality weights, producing a single cross-omics pathway importance.

To quantify explanation stability across K=5 folds, we compute a fold-specific pathway-importance vector $I^{\left(k\right)}={\left\{I_{p}^{\mathrm{int},(k)}\right\}}_{p}$ and report average pairwise Spearman correlation:

(9) $$\mathrm{Stability}=\frac{2}{K(K-1)}\sum_{1\leq i<j\leq K}\rho_{s}\left(I^{\left(i\right)},I^{\left(j\right)}\right).$$

A higher value indicates that the pathway ranking is more reproducible across cross-validation folds.

To increase confidence that attention-weighted explanations are not artefacts, we perform a label-permutation test: training on permuted labels should reduce predictive performance to near-chance and disrupt pathway rankings.

To quantify the additional pathway resolution enabled by the deeper masked hierarchy in AttentioFuse-5X, we summarize the attribution distribution over pathway nodes at each masked layer. Let $\left\{w_{i}\right\}$ denote the non-negative node importances for a given layer (aggregated across modalities when applicable) and $p_{i}=w_{i}/\sum_{j}w_{j}$ the normalized attribution mass. We report (i) the number of non-negligible nodes $N_{\geq \tau }=\#\left\{i:p_{i}\geq \tau \right\}$ with τ=0.001, and (ii) the effective number of contributing nodes $N_{\mathrm{eff}}=\exp\;\left(-\sum_{i}p_{i}\log p_{i}\right)$. These structural metrics characterize how many pathway nodes are meaningfully resolved at deeper Reactome levels, independent of predictive performance.

These metrics summarize how many pathway nodes meaningfully carry attribution mass, providing a quantitative proxy for biological resolution.

### 2.3. Comparative Model Implementation

We implemented classical and neural baselines: logistic regression (L2) [31], LASSO [32], Elastic-Net [33], Gaussian Naive Bayes [34], random forest [35], XGBoost [36], and a multilayer perceptron (MLP). The MLP mirrors the hidden-layer configuration used in our models (three fully connected layers with 256 units, LeakyReLU α=0.01, 30% dropout).

All experiments were evaluated using stratified 5-fold cross-validation. To prevent data leakage, preprocessing (scaling and any fold-wise imputation) was fitted on the training split of each fold only and then applied to the corresponding validation split using training-fold statistics. When enabled, Borderline-SMOTE was applied within the training split only.

We did not perform data-driven hyperparameter optimization (e.g., grid/random search) for the reported benchmarks. Instead, each model was evaluated with a fixed, pre-specified configuration or standard library defaults where appropriate, kept constant across folds and tasks (Table 1). This design avoids optimistic bias introduced by tuning on validation data and enables direct comparability across models. Accordingly, performance is reported as mean ± SD across the five folds.

### 2.4. Model Training Protocol

Optimization uses AdamW (Adam with decoupled weight decay) [37] (initial learning rate 0.01) with linear warm-up and Reduce-on-Plateau scheduling (factor 0.5, patience 5). Global gradient-norm clipping (L2-norm = 1.0) mitigates gradient explosions. Early stopping halts training after 10 consecutive epochs without validation loss improvement. Implementation used an NVIDIA RTX 4090 GPU with batch size 128. All linear layers were initialized with Kaiming (He) initialization [38]. Both 3F and AttentioFuse-5X are trained end-to-end under the same protocol.

## 3. Results

### 3.1. Introductory Summary of Omics-Model Characteristics

TNM staging was primarily evaluated on SMOTE-balanced datasets with oversampling confined to training folds inside stratified 5-fold CV. Accuracy (ACC) and weighted F1-score (F1) were used as headline metrics. The 3F model served as the main predictive backbone, whereas the 5X configuration was reserved for hierarchical interpretation. Under the balanced protocol, 3F and 5X exhibited highly similar trends (Supplementary Material File S2).

Under SMOTE balancing, M-stage classification reached near-ceiling performance across models. The near-ceiling performance for M-stage (especially in LUSC) is consistent with strong class separability and/or limited label complexity under the adopted M0 vs. M1 binarization. In TCGA, M1 cases are comparatively rare, and oversampling within training folds can further simplify discrimination by amplifying a small set of highly predictive molecular signatures. As a result, multiple model families achieve similarly high ACC/F1 and M-stage becomes less informative for comparing model capacity. We therefore focus mechanistic interpretation primarily on T- and N-stages, where discrimination is more challenging and pathway-level explanations are more diagnostic. For T-stage and N-stage, AttentioFuse-3F consistently matched or outperformed neural baselines and tree/boosting methods (Table 2). To mitigate concerns about oversampling artefacts, 3F without SMOTE was also evaluated under the same CV protocol; observed changes were small and task-dependent, supporting the SMOTE-balanced ACC/F1 as primary readouts. All metrics are summarized as mean ± SD across folds to reflect variability and robustness.

### 3.2. Quantitative Comparison Between 3F and 5X

To support the positioning of AttentioFuse-5X as an interpretation-oriented variant, we compared 3F and 5X in predictive quality under the same task definitions. The 3F configuration is reported with 5-fold cross-validation (mean ± SD; Table 2) as our main benchmarking backbone. The 5X configuration was evaluated in a dedicated interpretability-oriented setting to verify that additional depth does not materially degrade discrimination, while enabling deeper pathway-level analyses. As summarized in Table 3, 5X achieves comparable F1-scores on both LUAD and LUSC across TNM endpoints, supporting its use primarily for hierarchical interpretation rather than performance gains.

Beyond predictive performance (Table 3), we quantified the biological resolution enabled by the additional masked hierarchy in AttentioFuse-5X using the pathway-node attribution distributions underlying the Sankey visualizations (Supplementary Material File S3). In the two deeper layers unique to 5X, the intermediate layer (Layer 3; 153 nodes) resolved 50–110 non-negligible pathway nodes across tasks, with $N_{\mathrm{eff}}$ ranging from 11.30 to 85.97, while the top pathway layer (Layer 4; 29 nodes) retained 19–29 non-negligible nodes with $N_{\mathrm{eff}}$ ranging from 4.54 to 11.27. These results support that 5X provides additional hierarchical pathway structure at deeper Reactome levels, whereas 3F cannot expose these levels by design.

### 3.3. Interpretability Analysis of LUAD and LUSC Staging Characteristics

Unless otherwise noted, interpretability analyses were conducted with the five-layer AttentioFuse-5X variant, while predictive benchmarking relied on the 3F configuration. Attention maps and Sankey diagrams were derived by aggregating DeepSHAP gene attributions with global attention weights across cross-validation folds; pathway-level scores were normalized within stage.

#### 3.3.1. Validated Pathways and Novel Mechanisms in LUSC

**Figure 3:**
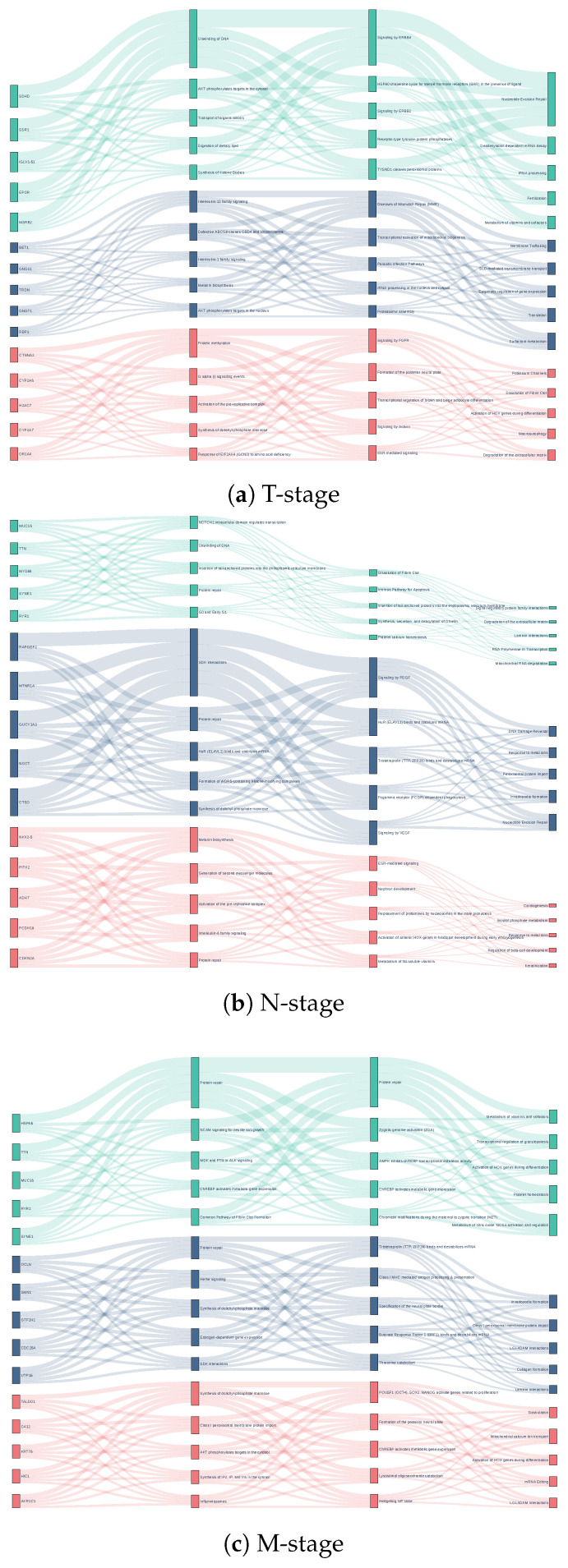
Sankey diagram visualization of key pathway contributions to TNM staging in LUSC. Nodes are colored by omics type: transcriptome (red), CNV (blue), and SNV (green). The diagram highlights validated mechanisms such as Hippo–Notch signaling (T-stage), PDGFR/VEGF-metalloregulation (N-stage), and ERBB–HSP90 coupling (M-stage), alongside putative novel findings including developmental pathway reactivation and microbiota-associated metastasis.

The framework demonstrated robust alignment with canonical squamous carcinoma biology while proposing testable hypotheses for LUSC progression (Figure 3). Key validated mechanisms included Hippo–Notch signaling crosstalk (T-stage), PDGFR/VEGF-linked metalloregulation (N-stage) [39], and ERBB–HSP90 functional coupling (M-stage), consistent with established LUSC molecular programs and therapeutic targets.

Putative novel signals centered on developmental pathway reactivation [40] (including germ-layer formation programs) and microbiota-associated metastasis [41], with signatures suggestive of parasite-infection–mitochondrial crosstalk. An unexpected melanogenesis signal [42] may reflect ROS regulation beyond pigmentation. In addition, WDR5–FCGR epigenetic linkages implied macrophage polarization via histone modification [43], warranting experimental validation.

To quantify the pathway-level structure summarized in Figure 3, we analyzed the normalized attribution distribution over the top Reactome pathway layer (Layer 4; 29 nodes) that underlies the Sankey visualization (Supplementary Material File S3). In LUSC T-stage, the top five pathways account for 78.7% of the attribution mass (top-10: 93.6%), dominated by Hemostasis (22.3%), Signal Transduction (17.9%), and Cell Cycle (16.3%). In N-stage, the top five account for 71.9% (top-10: 89.3%), led by Digestion and Absorption (27.6%) and Hemostasis (20.9%). In M-stage, the top five account for 69.3% (top-10: 89.2%), with Developmental Biology (21.6%) and Circadian Clock (18.9%) among the dominant contributors. Consistently, the effective number of contributing pathways at Layer 4 remains non-trivial (T: $N_{\mathrm{eff}}=9.16$, N: 10.21, M: 11.27), indicating a concentrated yet multi-module explanatory structure rather than diffuse, low-specificity attribution.

#### 3.3.2. Validated Pathways and Novel Mechanisms in LUAD

For adenocarcinoma, the model recapitulated mTORC1-mediated metabolic control in T-stage [44] and AKT–VEGF-linked angiogenic coupling in M-stage [45], aligning with LUAD metabolic and vascular dependencies. Putative novel hypotheses included embryonic nonsense-mediated mRNA decay activation [46] potentially sustaining stemness, and leptin signaling [47] consistent with obesity-associated matrix remodeling.

**Figure 4:**
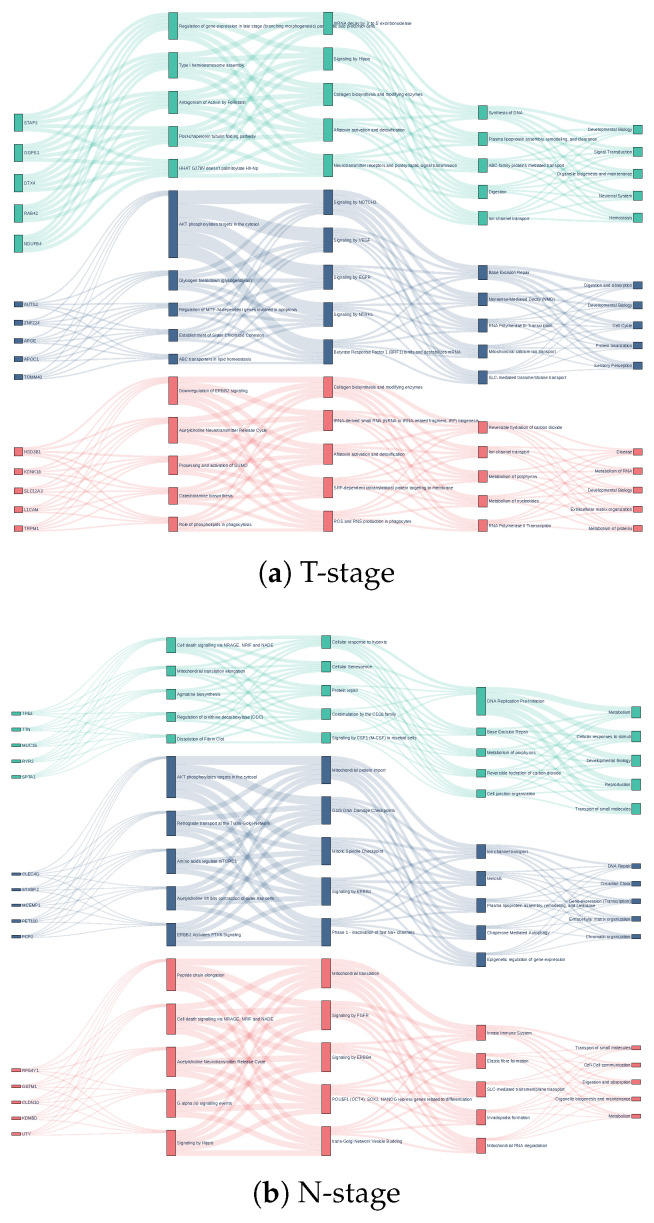

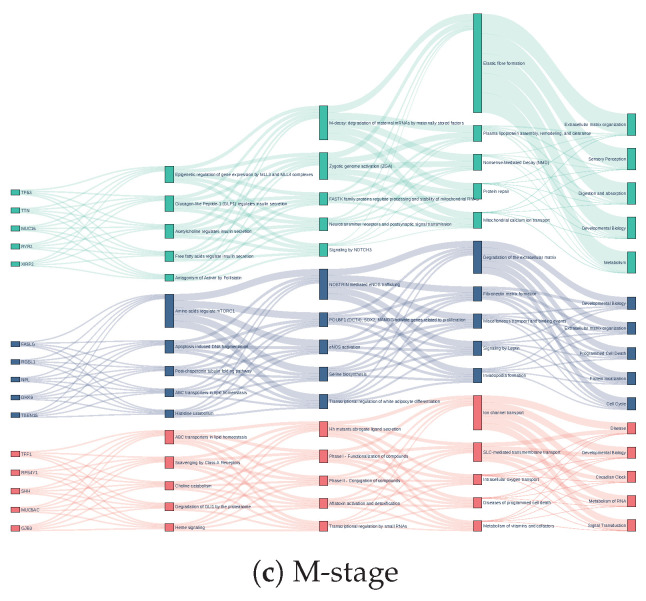
Sankey diagram of validated and putative novel pathway contributions in LUAD. The diagram highlights validated mechanisms such as mTORC1-mediated metabolic control in T-stage and AKT–VEGF angiogenic coupling in M-stage, alongside additional pathway hypotheses including nonsense-mediated mRNA decay, leptin signaling, mitochondrial calcium programs, collagen–Hedgehog crosstalk, and tRNA-derived networks.

Mitochondrial calcium–FASTK interactions [48] suggested ion-flux-regulated RNA stability as a metabolic plasticity mechanism. Metastatic programs further implicated non-canonical collagen–Hedgehog crosstalk [49] linking extracellular mechanics to post-translational regulation. Finally, tRNA-derived networks [50] supported a potential epigenetic–metastatic coupling axis, consistent with TIMP3-linked invasion control (Figure 4).

We similarly quantified the pathway-level attribution structure summarized in Figure 4 using the normalized Layer 4 distribution (29 nodes; Supplementary Material File S3). LUAD T-stage shows a highly concentrated profile, with the top five pathways explaining 96.1% of the mass (top-10: 97.9%), dominated by Developmental Biology (49.8%) and Extracellular Matrix Organization (21.1%). In contrast, LUAD N- and M-stages exhibit broader multi-module support: for N-stage, the top five pathways account for 73.0% (top-10: 88.8%), led by Metabolism (40.6%); for M-stage, the top five pathways account for 77.2% (top-10: 91.5%), with Developmental Biology (34.0%), Disease (16.4%), and Signal Transduction (13.4%) among the leading contributors. The corresponding effective numbers further reflect this stage dependence (T: $N_{\mathrm{eff}}=4.54$ vs. N: 9.52 and M: 9.14), providing a quantitative basis for the differing degrees of pathway concentration observed in the Sankey summaries.

### 3.4. NSCLC Common Mechanisms and Personalized Therapeutic Implications

Analyses revealed conserved oncogenic circuitry across LUAD and LUSC while highlighting histology-specific vulnerabilities. The AKT/mTOR axis emerged as a pan-NSCLC regulator across stages. Notch signaling exhibited histology-divergent roles; γ-secretase inhibitors combined with anti-angiogenics may warrant hypothesis-driven evaluation. Shared extracellular matrix remodeling signatures suggested potential liquid biopsy correlates. These histology-specific differences are summarized in Table 4, while modality-specific attention contributions are reported in Table 5; the full cross-omics, feature-level, and fusion-layer attention analyses are provided in Supplementary Material Figure S1.

## 4. Discussion

AttentioFuse demonstrates that model transparency can be achieved without sacrificing predictive performance in NSCLC multi-omics analysis. With Reactome-constrained encoders and a hierarchical attention stack, attributions are read coherently across levels—genes, pathways, and modalities—using DeepSHAP-based gradients. The 3F variant remains the primary predictive backbone due to its favorable bias–variance trade-off, while the 5X variant provides deeper pathway resolution with similar discrimination.

Several limitations should be considered when interpreting the present findings. First, interpretability is bounded by pathway prior coverage and curation quality; signals outside Reactome annotations may be under-represented. Second, TNM labels are workflow-dependent and may contain noise; our binarized endpoints reduce granularity-driven variance but cannot eliminate label uncertainty. Third, cohort size and class imbalance (notably for M1) can lead to near-ceiling performance and reduces the value of M-stage for model comparison. Fourth, attention gates and linear masks mainly capture approximately linear interactions within each attention slice, potentially under-modeling higher-order effects when modalities are sparse or missing. Finally, external evaluations are feasibility checks under modality-available settings; domain shift across cohorts (assay pipelines, cohort composition, and missing modalities) limits direct comparability and motivates future prospective validation on clinically collected multi-omics biopsies with harmonized protocols.

Importantly, the proposed explanations are hypothesis-generating: they highlight associations consistent with pathway priors and model attributions but do not imply causal effects without experimental validation.

From a translational perspective, AttentioFuse is intended for pre-treatment settings where diagnostic biopsies can be profiled (e.g., targeted sequencing and transcriptomics) to complement imaging-based staging. The model output can serve as a decision-support signal to prioritize intensified staging work-up (e.g., additional imaging or nodal assessment), support multidisciplinary planning, and provide pathway-resolved hypotheses that may guide biomarker-focused discussion of neoadjuvant/adjuvant strategies. The framework is not a replacement for pathology; rather, it provides mechanism-linked risk stratification that can be audited and interpreted alongside clinical findings.

## 5. Conclusions

AttentioFuse provides an interpretable, Reactome-guided mid-fusion framework for multi-omics staging prediction in LUAD and LUSC. By combining pathway-masked encoders with hierarchical attention and DeepSHAP-based attribution, the framework links predictive signals to a coherent gene–pathway–modality evidence chain.

Across TCGA cohorts, AttentioFuse achieves competitive discrimination for T- and N-stage and near-ceiling performance for M-stage under a binarized endpoint definition. The dual-depth design enables practical trade-offs: the 3F configuration serves as a robust benchmarking backbone, whereas the 5X configuration increases hierarchical resolution for interpretation while maintaining comparable predictive quality.

Future work should focus on prospective validation in clinically collected biopsy cohorts with harmonized pipelines and on extending the modality-available protocol to broader real-world settings. Importantly, the reported pathway findings are hypothesis-generating and are intended to prioritize mechanistic follow-up rather than imply causal inference.

## Figures and Tables

**Table 1:** Fixed hyperparameter configurations used for model comparison. No data-driven hyperparameter optimization was performed.

Model	Configuration (Fixed Across Folds/Tasks)
AttentioFuse (3F/5X)	AdamW; initial lr = 0.01; batch size = 128; early stopping (patience = 10); gradient clipping (1.0); Reduce-on-Plateau (factor = 0.5, patience = 5); Kaiming initialization; LeakyReLU (α = 0.01).
IntegratalNet/MLP	Same cross-validation protocol; MLP with 3 hidden layers (256 units), LeakyReLU (α = 0.01), dropout = 0.30.
LogReg (L2)	Standard implementation with fixed regularization strength; stratified 5-fold CV.
LASSO/Elastic-Net	Standard implementation with fixed regularization strength (and fixed $l_{1}$ ratio for Elastic-Net).
Random Forest/XGBoost	Standard implementations with default settings.
GaussianNB	Default settings.

**Table 2:** Comparative performance of multi-model approaches in TNM staging prediction for LUAD and LUSC cohorts with Borderline-SMOTE. Values are reported as mean ± SD across 5 folds (ACC and weighted F1).

Cohort	Stage	IntegratalNet	AttentioFuse-3F	MLP	LogReg	XGBoost	LASSO	Elastic-Net	GNB	RF
LUAD	T	ACC: 0.81 ± 0.06 F1: 0.80 ± 0.03	ACC: 0.89 ± 0.07 F1: 0.89 ± 0.08	ACC: 0.73 ± 0.17 F1: 0.73 ± 0.12	ACC: 0.59 ± 0.03 F1: 0.66 ± 0.03	ACC: 0.87 ± 0.01 F1: 0.81 ± 0.02	ACC: 0.68 ± 0.03 F1: 0.73 ± 0.03	ACC: 0.59 ± 0.04 F1: 0.66 ± 0.03	ACC: 0.87 ± 0.00 F1: 0.81 ± 0.00	ACC: 0.87 ± 0.01 F1: 0.82 ± 0.01
	N	ACC: 0.56 ± 0.12 F1: 0.53 ± 0.19	ACC: 0.87 ± 0.15 F1: 0.87 ± 0.16	ACC: 0.55 ± 0.16 F1: 0.49 ± 0.17	ACC: 0.60 ± 0.05 F1: 0.61 ± 0.05	ACC: 0.66 ± 0.04 F1: 0.64 ± 0.04	ACC: 0.60 ± 0.05 F1: 0.61 ± 0.05	ACC: 0.60 ± 0.05 F1: 0.60 ± 0.05	ACC: 0.64 ± 0.02 F1: 0.53 ± 0.03	ACC: 0.70 ± 0.03 F1: 0.66 ± 0.03
	M	ACC: 0.92 ± 0.01 F1: 0.89 ± 0.01	ACC: 0.94 ± 0.02 F1: 0.92 ± 0.03	ACC: 0.91 ± 0.01 F1: 0.89 ± 0.00	ACC: 0.60 ± 0.00 F1: 0.70 ± 0.00	ACC: 0.93 ± 0.00 F1: 0.90 ± 0.00	ACC: 0.74 ± 0.04 F1: 0.80 ± 0.02	ACC: 0.61 ± 0.02 F1: 0.70 ± 0.02	ACC: 0.93 ± 0.00 F1: 0.90 ± 0.00	ACC: 0.93 ± 0.00 F1: 0.90 ± 0.00
LUSC	T	ACC: 0.74 ± 0.06 F1: 0.71 ± 0.03	ACC: 0.81 ± 0.11 F1: 0.82 ± 0.10	ACC: 0.72 ± 0.11 F1: 0.70 ± 0.07	ACC: 0.59 ± 0.03 F1: 0.64 ± 0.02	ACC: 0.79 ± 0.03 F1: 0.73 ± 0.04	ACC: 0.65 ± 0.03 F1: 0.68 ± 0.03	ACC: 0.59 ± 0.03 F1: 0.64 ± 0.02	ACC: 0.82 ± 0.00 F1: 0.74 ± 0.00	ACC: 0.82 ± 0.01 F1: 0.74 ± 0.00
	N	ACC: 0.55 ± 0.11 F1: 0.50 ± 0.13	ACC: 0.78 ± 0.15 F1: 0.77 ± 0.16	ACC: 0.61 ± 0.03 F1: 0.60 ± 0.04	ACC: 0.58 ± 0.04 F1: 0.59 ± 0.05	ACC: 0.63 ± 0.04 F1: 0.60 ± 0.05	ACC: 0.60 ± 0.04 F1: 0.60 ± 0.04	ACC: 0.57 ± 0.04 F1: 0.57 ± 0.04	ACC: 0.65 ± 0.02 F1: 0.53 ± 0.03	ACC: 0.62 ± 0.03 F1: 0.57 ± 0.05
	M	ACC: 0.98 ± 0.01 F1: 0.98 ± 0.00	ACC: 0.98 ± 0.01 F1: 0.98 ± 0.00	ACC: 0.98 ± 0.01 F1: 0.98 ± 0.00	ACC: 0.68 ± 0.00 F1: 0.80 ± 0.00	ACC: 0.99 ± 0.00 F1: 0.98 ± 0.00	ACC: 0.93 ± 0.02 F1: 0.95 ± 0.01	ACC: 0.71 ± 0.03 F1: 0.81 ± 0.02	ACC: 0.99 ± 0.00 F1: 0.98 ± 0.00	ACC: 0.99 ± 0.00 F1: 0.98 ± 0.00

**Table 3:** Quantitative comparison between AttentioFuse-3F and AttentioFuse-5X under the same stratified 5-fold CV protocol. Values are mean ± SD (ACC/F1).

Cohort	Task	Variant	ACC (Mean ± SD)	F1 (Mean ± SD)
LUAD	T	3F	0.89 ± 0.07	0.89 ± 0.08
LUAD	T	5X	0.92 ± 0.04	0.95 ± 0.06
LUAD	N	3F	0.87 ± 0.15	0.87 ± 0.16
LUAD	N	5X	0.73 ± 0.16	0.76 ± 0.18
LUAD	M	3F	0.94 ± 0.02	0.92 ± 0.03
LUAD	M	5X	0.99 ± 0.00	0.99 ± 0.00
LUSC	T	3F	0.81 ± 0.11	0.82 ± 0.10
LUSC	T	5X	0.96 ± 0.02	0.93 ± 0.00
LUSC	N	3F	0.78 ± 0.15	0.77 ± 0.16
LUSC	N	5X	0.80 ± 0.15	0.80 ± 0.10
LUSC	M	3F	0.98 ± 0.01	0.98 ± 0.00
LUSC	M	5X	0.99 ± 0.00	1.00 ± 0.00

**Table 4:** Histology-specific divergences.

Feature	LUAD Signature	LUSC Signature
Driver alterations	EGFR/KRAS/ALK mutations	PIK3CA/SOX2 amplifications
Metabolic reprogramming	CPT1A-driven fatty acid oxidation dominance	HK2/PKM2-driven glycolytic flux amplification
Immune landscape	CD68-marked PD-L1^+^ TAM-enriched microenvironment	CD8A-marked CD8^+^ T-cell infiltrated microenvironment
Metastatic pattern	Hematogenous (bone/brain) via AKT–VEGF angiogenesis	Locoregional (mediastinum) via Hippo–Notch crosstalk

**Table 5:** Contribution scores across the three attention mechanisms within AttentioFuse-5X.

Cohort	Modality	Cross-Omics			Feature-Level			Fusion-Layer		
		T	N	M	T	N	M	T	N	M
LUAD	SNV	0.17	0.58	0.62	0.10	0.55	0.10	352.44	1969.28	363.14
	CNV	–	–	–	0.10	0.54	0.10	356.30	1935.07	367.40
	Transcriptome	–	–	–	0.10	0.54	0.10	360.97	1963.64	352.20
LUSC	SNV	0.16	0.19	0.21	0.12	0.12	0.12	514.42	509.82	515.76
	CNV	–	–	–	0.12	0.12	0.12	518.30	512.27	527.57
	Transcriptome	–	–	–	0.12	0.12	0.12	521.04	515.37	517.99

## Data Availability

The source code for the AttentioFuse framework is available on GitHub under the MIT license: https://github.com/YuHang-aw/Attentio_Fuse.git (accessed on 5 March 2026). Public LUAD and LUSC data from TCGA were accessed with TCGAbiolinks and are also available from the GDC portal https://portal.gdc.cancer.gov/ (accessed on 15 May 2025). The Reactome pathway database (v86) can be downloaded from https://reactome.org/download-data (accessed on 10 May 2025).

## Supplementary Materials

The following supporting information can be downloaded at: https://www.mdpi.com/article/10.3390/cancers18050878/s1, Supplementary Material File S1: External portability analyses on ICGC and GTEx under the modality-available protocol; Supplementary Material File S2: Full benchmarking results for all evaluated models and modalities; Supplementary Material File S3: Feature-importance scores (gene and pathway levels); Supplementary Material Figure S1: Attention-contribution analyses (cross-omics, feature-level, fusion-layer components).
